# Implementing a pharmacist-integrated collaborative model of medication treatment for opioid use disorder in primary care: study design and methodological considerations

**DOI:** 10.1186/s13722-024-00452-y

**Published:** 2024-03-18

**Authors:** Bethany McLeman, Phoebe Gauthier, Laurie S. Lester, Felicity Homsted, Vernon Gardner, Sarah K. Moore, Paul J. Joudrey, Lisa Saldana, Gerald Cochran, Jacklyn P. Harris, Kathryn Hefner, Edward Chongsi, Kimberly Kramer, Ashley Vena, Rebecca A. Ottesen, Tess Gallant, Jesse S. Boggis, Deepika Rao, Marjorie Page, Nicholas Cox, Michelle Iandiorio, Ekow Ambaah, Udi Ghitza, David A. Fiellin, Lisa A. Marsch

**Affiliations:** 1 Northeast Node, NIDA Drug Abuse Treatment Clinical Trials Network, Hanover, NH USA; 2 Center for Technology and Behavioral Health, Geisel School of Medicine at Dartmouth College, Hanover, NH USA; 3 FQHC 340B Compliance, Lebanon, TN USA; 4 University of Pittsburgh School of Medicine, Pittsburgh, PA USA; 5 Lighthouse Institute, Chestnut Health Systems, Eugene, OR USA; 6 University of Utah, Salt Lake City, UT USA; 7 Greater Intermountain Node, NIDA Drug Abuse Treatment Clinical Trials Network, Salt Lake City, UT USA; 8 The Emmes Company, LLC, Rockville, MD USA; 9 Community Health Care, Tacoma, WA USA; 10 University of Utah College of Pharmacy, Salt Lake City, UT USA; 11 University of New Mexico School of Medicine, Albuquerque, NM USA; 12 Harbor Care Health & Wellness, Nashua, NH USA; 13 National Institute on Drug Abuse, North Bethesda, MD USA; 14 New England Consortium Node, NIDA Drug Abuse Treatment Clinical Trials Network, New Haven, CT USA; 15 Program in Addiction Medicine, Department of Medicine, Yale School of Medicine, New Haven, CT USA

**Keywords:** Pharmacists, Evidence-based pharmacy practice, Opioid use disorder, Implementation science, Feasibility studies, Medications for opioid use disorder, Buprenorphine

## Abstract

**Background:**

Pharmacists remain an underutilized resource in the treatment of opioid use disorder (OUD). Although studies have engaged pharmacists in dispensing medications for OUD (MOUD), few studies have evaluated collaborative care models in which pharmacists are an active, integrated part of a primary care team offering OUD care.

**Methods:**

This study seeks to implement a pharmacist integrated MOUD clinical model (called PrIMO) and evaluate its feasibility, acceptability, and impact across four diverse primary care sites. The Consolidated Framework for Implementation Research is used as an organizing framework for study development and interpretation of findings. Implementation Facilitation is used to support PrIMO adoption. We assess the primary outcome, the feasibility of implementing PrIMO, using the Stages of Implementation Completion (SIC). We evaluate the acceptability and impact of the PrIMO model at the sites using mixed-methods and combine survey and interview data from providers, pharmacists, pharmacy technicians, administrators, and patients receiving MOUD at the primary care sites with patient electronic health record data. We hypothesize that it is feasible to launch delivery of the PrIMO model (reach SIC Stage 6), and that it is acceptable, will positively impact patient outcomes 1 year post model launch (e.g., increased MOUD treatment retention, medication regimen adherence, service utilization for co-morbid conditions, and decreased substance use), and will increase each site’s capacity to care for patients with MOUD (e.g., increased number of patients, number of prescribers, and rate of patients per prescriber).

**Discussion:**

This study will provide data on a pharmacist-integrated collaborative model of care for the treatment of OUD that may be feasible, acceptable to both site staff and patients and may favorably impact patients’ access to MOUD and treatment outcomes.

*Trial registration:* The study was registered on Clinicaltrials.gov (NCT05310786) on April 5, 2022, https://www.clinicaltrials.gov/study/NCT05310786?id=NCT05310786&rank=1

## Background

Across the US, the prevalence of opioid use disorder (OUD) and the rates of opioid overdoses have risen precipitously in recent years. Overdose deaths in the US increased 135% from 2013 to 2021 (13.8 to 32.4 age-adjusted overdose death rate) [1]. Between March–August of 2020, in the midst of the COVID-19 global pandemic, overdose deaths exceeded pre-pandemic levels, with 10,443 more deaths than predicted [2]. Effective FDA-approved medications for the treatment of OUD (MOUD) include buprenorphine, methadone, and naltrexone, but less than half of people with OUD engage or remain in treatment long enough to realize benefits and reduce the risk of overdose [3, 4]. In 2021, about one-third (35.6%) of people needing OUD treatment in the US reported having received MOUD [5]. The severity of the opioid crisis and recognition of these gaps in treatment have led to a call for an “all hands-on deck” approach to scaling-up delivery of MOUD [6].

As “today’s medication-use leaders” [7], pharmacists are well-positioned to extend their role beyond offering consultative services to engaging in integrated approaches to addressing patients’ medication needs across a care continuum and reducing the gaps in care for OUD. While pharmacists in the US and abroad make important contributions such as prescription opioid screening, prescription management, patient education (including brief educational interventions), and treatment referrals [8–11], practice models that fully incorporate and leverage these capabilities are underdeveloped. Although studies have engaged pharmacists in dispensing naloxone, syringes, MOUD, or MOUD with limited pharmacist-physician communication [12, 13], few studies have evaluated collaborative care models in which pharmacists are integrated into the OUD care team [14].

One particularly promising model of pharmacist-integrated care for OUD, the Pharmacist-Integrated Medication Treatment for OUD (PrIMO) model, was launched in 2017 by an interdisciplinary team led by pharmacist Dr. Felicity Homsted and psychiatrist Dr. Vernon “Trip” Gardner at Penobscot Community Health Care (PCHC; the largest system of federally qualified health centers in Maine) [15, 16]. The PrIMO model reflects an ambulatory care pharmacy practice model [17]; an integrated primary care clinical team including physicians, psychiatrists, nurse practitioners, behavioral health clinicians, and pharmacy staff (including technicians) work collaboratively to provide healthcare to persons with OUD. In this model, the pharmacist’s role expands beyond existing duties to include direct engagement in all team activities as an embedded care team member. The PrIMO model has shown promise and was associated with improved patient engagement with OUD treatment and increased receipt of services for co-morbid chronic diseases, housing and employment needs [16]. After the launch of PrIMO, PCHC increased the number of clinicians prescribing MOUD (from 4 to 24) and increased pharmacy staff’s attitudes toward addiction treatment [16]. Prescribers in family practice settings reported that this enhanced level of support helped decrease their concerns about delivering MOUD. Additionally, prescribers reported that the model improved their confidence in prescribing MOUD within primary care instead of referring patients to specialty settings. Within 1 year of launch, this model was implemented in two additional PCHC health centers. In collaboration with other efforts made at the clinic, the PrIMO model helped reduce the number of patients on long-term opioids, long-term benzodiazepines, and those receiving more than 100 daily morphine milligram equivalents of opioids.

The PrIMO model is reflective of an “ambulatory care pharmacy practice” which was proposed by a joint task force of the American Pharmacists Association, the American Society of Health-System Pharmacists, and the American College of Clinical Pharmacy [17]. Ambulatory care pharmacists provide comprehensive medication management (CMM), which is designed to optimize medication use to improve outcomes using a patient-centered approach [18–20] to chronic health conditions and improve medication safety [21]. It has been noted that when pharmacists are engaged in patient care in this manner, physician time is saved, access to care is improved and clinical and economic outcomes are significantly improved [17, 22]. There is evidence that this approach may be viewed by payers as financially viable [23], particularly alongside other value based care models [24, 25]. Additionally, given the growing supply of pharmacists in the US (e.g., the number of schools of pharmacy in the US has almost doubled since 2000 and pharmacist supply is expected to grow by 35% by 2025) [26–29], pharmacists with specialized training may be increasingly available to participate in these integrated models of care. However, ambulatory pharmacy care integration can be limited due to operational constraints and policies of the clinic and/or pharmacy organizations [30]. Given that integrated clinical teams have been among the most successful models of care for chronic diseases, and embedding pharmacists for chronic disease management has proven effective, an urgent next step is to examine this approach with the inclusion of pharmacists on MOUD care teams [31, 32].

### Rationale for study design

This paper describes the protocol, design considerations, and methods for a multi-site study examining implementation of the PrIMO model in four diverse primary care settings. As noted above, the PrIMO model was rooted in practices used to manage other chronic conditions (e.g., CMM and ambulatory pharmacy care) and was adopted as the clinical model for MOUD at several of PCHC’s primary care clinics. The present study provides the opportunity to understand the feasibility of implementing PrIMO in a diverse array of other settings outside of PCHC, including sites in different regions of the US and with different operational structures than PCHC. The study will also enable an assessment of the acceptability and impact of implementing PrIMO in these diverse sites. This study will additionally allow for the opportunity to learn about the process by which diverse sites may implement the model and the site-specific barriers and facilitators to doing so. Supported by the National Drug Abuse Treatment Clinical Trials Network (CTN) within the National Institute on Drug Abuse (NIDA), the project (CTN-0116) is grounded in implementation science and leverages the Consolidated Framework for Implementation Research (CFIR) [33], a widely used determinant framework, and supporting procedures (i.e., implementation assessment strategies) from Implementation Facilitation (IF) [34] to evaluate the feasibility, acceptability, and impact of the PrIMO model in four diverse primary care settings.

## Methods

### Overview

We will assess the primary outcome, the ability to achieve successful implementation of PrIMO, using the Stages of Implementation Completion (SIC) [35]. We will evaluate the acceptability and impact of the PrIMO model at the sites using mixed-methods and combine survey and CFIR-informed interview data from providers, pharmacists, pharmacy technicians, administrators, and patients receiving MOUD at the primary care sites with patient electronic health record data. The study protocol was developed and reviewed by a Data Safety and Monitoring Board (DSMB) convened by the NIDA CTN. The Emmes Company serves as the Clinical Research Organization (CRO), providing data and statistical support and clinical coordinating services. This study was reviewed and approved by the Biomedical Research Alliance of New York (BRANY) Institutional Review Board. The study was registered on Clinicaltrials.gov (NCT05310786) on April 05, 2022.

#### The PrIMO model

In the study planning stage, PrIMO model developers (FH, VG) gathered a multidisciplinary team to operationalize active ingredients of the model’s effectiveness. Discussions centered on model conduct, team structure, and a non-judgmental approach to care. Engaging patients with OUD who have experience being cared for under the model at PCHC for first-hand feedback, consensus was reached on core components. The CFIR [33] guided consideration of intervention characteristics (e.g., innovation source, evidence base, design, and adaptability) likely to influence implementation in the design of an accompanying toolkit to support model adoption. The components of the PrIMO model were defined, and key components were identified and operationalized (see Table 1). In the PrIMO model, an interdisciplinary team of providers is brought together to treat OUD following the Collaborative Care model used with other chronic conditions. The pharmacist is a valued member of this care team and provides expertise of medications, dosing, insurance needs (e.g., prior authorizations), and consideration of state pharmacy scope of practice laws. During the model’s development, this pharmacist engagement allowed the prescribers to feel more confident about providing care to patients with OUD. In addition, encounters with patients filling MOUD prescriptions at the pharmacy can provide information to the care team related to the patient’s treatment. In these ways, the pharmacist is a key provider of MOUD services at the clinic under the PrIMO model. In this study, the PrIMO model is delivered by the care team at each site as a clinical procedure in conjunction with the research. Since the PrIMO model was designed to provide pharmacist-integrated care for OUD at an FQHC, minor adaptations will be made to fit the four primary care settings.

**Table 1:** Pharmacist-integrated medication treatment for OUD (PrIMO) core components

Core component	Description
Collaborative communication	Regular collaborative communication among all members of the care team is the cornerstone of this integrated model. Clinicians will communicate needs or questions about medications, and pharmacists will communicate information learned through other touch points of MOUD care (i.e., observations at the pharmacy when a patient picks up medication)
PrIMO operations team meetings	At least weekly, the clinical care team providing MOUD at the site will meet to discuss operations of the treatment program, specific patient needs, and clinic or workflow needs related to the PrIMO model
Support and educate providers	The pharmacist will support and educate providers and other site staff on a variety of topics that fall under their realm of expertise. For example, pharmacists may assist in the initiation to MOUD, supporting the provider in selecting the appropriate medication, dosing, and ongoing management
Support and educate patients	The pharmacist will offer to meet with patients one-on-one to discuss medications, concerns (including insurance coverage and needed prior authorizations), co-management of other health conditions alongside MOUD, and specifics of the medication prescribed
Screen for and manage comorbid conditions	The PrIMO team will develop or manage an existing health screening order that can include blood tests as well as clinical assessments for risk of many health conditions that patients with OUD often comanage. The pharmacist will work to address these comorbid conditions and their associated treatment, preventative measures for at-risk conditions (including immunizations and vaccinations), and coordinate referrals to specialty providers outside of the clinic. The pharmacist will have a large role in managing health screening and coordinating care across other care team members
Educate local partners	As integrated MOUD care providers, the PrIMO team at the site will be encouraged to share learning opportunities with other local partners at the clinic and/or within the local community

#### Implementation Facilitation strategy

Implementation Facilitation (IF) is the comprehensive strategy incorporating a bundle of interconnected implementation activities that will be used to support and enable clinical sites to implement the PrIMO model. The IF strategy utilized in this study was manualized by Kirchner and colleagues [34] and used by study investigators in previous studies [36–39]. In this study, IF relies on a collaborative partnership between local site champions (provider and pharmacist) who have in-depth knowledge of the organization (e.g., norms, culture, change agents) and external facilitators who serve as content experts on the innovation (PrIMO model developers, addiction and psychiatry expertise, implementation researchers). Champions serve as leaders of practice change within the clinic, forging relationships with local partners, tailoring the model to site-specific conditions, and building enthusiasm. They work closely with external facilitators and serve as key liaisons between the two teams. External facilitators work with local partners and provider and pharmacist champions to conduct a formative evaluation, develop, and refine clinical procedures or resources, perform academic detailing and staff education, lead a learning collaborative offering continuing medical education units, and facilitate performance monitoring and feedback. Elements of the IF approach are outlined in Table 2.

**Table 2:** Implementation Facilitation (IF) activities

IF activities	IF activity description
Engagement of local partners	Local partner engagement involves creating an atmosphere that is open, non-critical, and goal oriented. Engagement of key partners will take place at the administrative, provider, pharmacist, pharmacy technician, and patient levels
Formative evaluation	Formative evaluation is an implementation assessment approach designed to identify influences on the development, progress, and effectiveness of implementation efforts related to the PrIMO model and use these data to tailor, refine, and monitor the implementation of the model. Approximately two focus groups per site will be conducted with additional individual meetings with local partners. Change rulers will provide ongoing evaluation of readiness and preparedness, gathered electronically
Learning collaborative	The monthly Learning Collaborative will bring together clinic staff from all four sites to discuss model implementation, problem solve, and support sustainability
Academic detailing	Academic detailing, provided by the external facilitators, will include sharing a review of clinical evidence that supports the PrIMO model implementation with key site partners. All local partners involved in the PrIMO model will be offered educational sessions, specifically tailored to an individual’s tasks and identified needs
Activity logs	Activity tracking logs will document the IF activity occurring, the roles of the individuals participating, and identified resources from the activity. Additionally, sites will document a running list of barriers to implementation
Tailoring of PrIMO model to each site	The IF strategy will be tailored to the local site informed by the formative evaluation and with feedback from local champions
Performance monitoring and feedback	External facilitators will collaborate with clinics to develop an electronic dashboard to track key metrics related to model performance
Program marketing	External facilitators will collaborate with local champions to develop program marketing designed to increase awareness of the PrIMO model through various forms of communication and media

### Aims, design and setting

#### Study aims

The primary aim of the study is to evaluate the feasibility of implementing the PrIMO model using the IF implementation approach into the clinical workflow across four primary care clinical sites. The secondary aims are to evaluate the acceptability and impact of the PrIMO model across the four sites. We will explore the impact of site characteristics, barriers and facilitators to implementation, and/or data collected via study process measures that might explain or support findings of the primary and secondary analyses.

#### Design

This is a longitudinal mixed-methods implementation study that combines qualitative and quantitative methods using survey and EHR data. Qualitative and quantitative outcomes will be measured across sites, site staff participants (e.g., providers, pharmacists, pharmacy technicians, and administrators), and patient participants in MOUD to assess the extent of implementation and barriers and facilitators to implementation and sustainability.

Site staff must be 18 years of age or older, speak and read English fluently, employed by the participating clinic, and were exposed to the PrIMO model through an introductory email sent by the Site Principal Investigator to all staff (this email contained a letter from the model developers and a graphic explaining the six Core Components of the PrIMO model). They will be recruited via posters, emails, or word-of-mouth and after providing informed written consent will complete quantitative surveys at approximately 3 months pre-PrIMO launch, at launch, 3 months post-launch, 6 months post-launch, 9 months post-launch, and 12 months post-launch. Site staff will be invited to participate in qualitative interviews at 3 months pre-launch, at launch, 6 months post-launch, and 12 months post-launch timepoints. Site staff will have the opportunity to participate at each timepoint to best capture the model’s acceptability.

Patients must be 18 years of age or older, receiving MOUD from a provider at the participating clinic, and exposed to the model for at least 2 weeks (to best protect study data from contamination related to the clinic’s MOUD procedures prior to PrIMO launch). Patients will be recruited from the participating clinics via posters, flyers, and word-of-mouth; they complete baseline, 3-month post-baseline, and 6-month post-baseline surveys and will be invited to participate in interviews at each timepoint. Patient enrollment will last for approximately 6 months.

EHR data, including aggregate treatment outcomes from all patients on MOUD at the clinic, and administrative data, including data on provider capacity, MOUD prescribing, and staff training, will inform impact and be collected for a period of 12 months pre- and post-model launch.

The CFIR theoretical framework [33, 40] was used by the study team to guide the overall project and identify domains and constructs that may influence the implementation of the PrIMO model. The CFIR is used to frame the contextual determinants of implementation through five overarching domains (i.e., innovation, outer setting, inner setting, individuals, and implementation process) and more than 47 detailed constructs within those domains. The study includes an array of measures that align with CFIR constructs deemed most relevant. These a priori identified CFIR constructs were also used to develop qualitative data collection and analytic approaches (i.e., interview guide, codebook) and will be used as a guide for analyzing, interpreting, and reporting implementation-related findings. Additionally, many of the CFIR constructs are present throughout the IF strategy employed in this study.

#### Setting

A site selection survey and voting process designed specifically for the study and conducted using the nation CTN infrastructure was used to collect information about prospective primary care clinics to identify four diverse sites in which to conduct the study. Primary care clinics from across the US submitted surveys expressing interest in participating in the trial; of them, four were chosen to implement PrIMO. Each of the four clinics selected to participate in this study had diverse patient populations (e.g., gender, race, ethnicity, geography), at least one clinic-based pharmacist, at least one active MOUD prescriber, and a retail pharmacy (see Table 3 for an overview of the four participating study sites).

**Table 3:** Site characteristics overview

	Site A	Site B	Site C	Site D
Site location	New Hampshire	Utah	Washington	New Mexico
Area	Medicaid expansion Medicaid coverage for MOUD HRSA-designated shortage area	Medicaid expansion Medicaid coverage for MOUD	Medicaid expansion Medicaid coverage for MOUD HRSA-designated shortage area	Medicaid expansion Medicaid coverage for MOUD HRSA-designated shortage area
Primary care clinic type	FQHC	Hospital/medical center	FQHC	Hospital/medical center
Unique primary care patients	2681	19,265	9488 patients	1900
Unique MOUD patients	350	123	221	35
Unique primary care prescribers (any Rx)	4	26	54	9
Unique MOUD prescribers	5	4	48	7
MOUD options	Buprenorphine Buprenorphine/naloxone Extended-release buprenorphine Naltrexone Extended-release naltrexone	Buprenorphine Buprenorphine/naloxone Extended-release buprenorphine Naltrexone Extended-release naltrexone	Buprenorphine Buprenorphine/naloxone Extended-release buprenorphine Naltrexone Extended-release naltrexone	Buprenorphine Buprenorphine/naloxone Extended-release buprenorphine Naltrexone Extended-release naltrexone
Clinic characteristics	• Staffing includes MD, advanced practice practitioners, RN, behavioral health, social workers, or recovery coach presence • No DO, residents • Serves unhoused and formerly incarcerated people • Close collaboration approaching an integrated practice	• Staffing includes MD, Residents, advanced practice practitioners, RN, social work, and recovery coaching • No DO or behavioral health presence • Established referral system from other medical center departments (e.g., emergency) • Close collaboration approaching an integrated practice	• Staffing includes MD, DO, Residents, advanced practice practitioners, RN, behavioral health • MOUD patient coordinator • No recovery coach presence • Serves unhoused population • Full collaboration in a transformed/merged integrated practice	• Staffing includes MD, DO, Residents, advanced practice practitioners, RN, behavioral health, social work • No recovery coach presence • Serves transgender/non-binary, unhoused, and people in treatment for infectious disease • Full collaboration in a transformed/merged integrated practice
Pharmacy characteristics	• 19,789 prescriptions filled • 2 pharmacists, 2 pharmacy techs • 340B member • Retail pharmacy on-site • No established clinical pharmacist role	• 120,630 prescriptions filled • 5 pharmacists, 9 pharmacy techs • Ambulatory care pharmacy team includes 1 pharmacist and 1 technician • 340B member • Retail pharmacy on-site • Embedded clinical pharmacist in family and internal medicine • Offsite clinical manager oversees ambulatory care pharmacists, pharmacy resident, and embedded clinic pharmacy technician	• 41,599 prescriptions filled • 2 pharmacists, 2 pharmacy techs • 340B member • Retail pharmacy on-site/nearby • Clinical pharmacist sees patients with other chronic diseases (e.g., hypertension, diabetes) • Carries extended-release buprenorphine and HIV medications for specialty clinics onsite	• ~ 72,000 prescriptions filled • 4 pharmacists, 8 pharmacy techs • 340B member • Retail pharmacy on-site • Clinical pharmacist sees people with HIV, HepC, and those with metabolic issues • Pharmacists have prescriptive authority to prescribe and dispense Narcan; clinic-based extended-release buprenorphine

The four sites are diverse in several ways (i.e., location, size and composition of provider/pharmacist/patient populations, operational setting, and MOUD services offered) and provide ample opportunity to observe variations in the PrIMO model’s implementation.

### Study population

To assess feasibility, acceptability, and impact of the model, different stakeholder groups will form our study population. Each outcome will require a different sampling frame as described below.

#### Feasibility of implementing the PrIMO model using the SIC

Primary care clinics participating in this study will serve as the unit of observation for the primary outcome and will be tracked as they move through site-specific activities related to planning for model implementation, model launch, and achieving competency with PrIMO as the standard model of care for OUD.

#### Acceptability of the PrIMO model

Adult, English-speaking, individuals who are not incarcerated and who are willing to provide consent will contribute data in two distinct convenience sample groups: site staff and patients receiving MOUD treatment. Among the site staff, a range of site staff participants employed by the primary care clinic or pharmacy in one of four roles will be eligible: provider (5–20 per site; may include MD, DO, NP, PA, nurse, behavioral health providers, and/or other clinical roles that provide care to patients), pharmacist (2–6 per site), pharmacy technician (2–10 per site), or administrator (2–10 per site; may include CEO, CFO, CMO, clinic managers, front desk staff, and/or other administrative roles that oversee programming at the clinic). A subset of site staff participants (up to 11 per site) also will be invited to participate in qualitative interviews. Site staff participants will be recruited for qualitative and quantitative measurements. Patient participants (12–40 per site) will also be English-speaking, not prisoners, willing and able to provide consent, and be receiving MOUD from the participating clinic and exposed to the model for at least 14 days to be eligible for the study. A subset of patient participants (up to 12) will be invited to participate in qualitative interviews. Patient participants will be recruited on an ongoing basis for a period of 6 months post-PrIMO launch.

#### Impact of the PrIMO model

EHR data from each site will be extracted throughout the study period for all adult patients receiving MOUD. Extracted EHR data will span 1 year pre-model launch and 1-year post-model launch to explore impact at each clinic.

### Processes and measures

#### Feasibility

Feasibility of launching the PrIMO model will be assessed using the SIC. The SIC is an eight-stage tool that tracks implementation processes and milestones, with stages spanning three implementation phases: pre-implementation (stages 1–3), implementation (stages 4–7), and competency (stage 8) [41–49]. The SIC is a date-driven measure with item responses including the date by which implementation activities are completed. The SIC yields three scores: (1) Duration—time to complete implementation activities, (2) Proportion—percent of activities completed, and (3) Final stage—the furthest point in the implementation process achieved. The SIC also gathers project-specific, site-level characteristics identified a priori that may impact implementation. This tool has been adapted for many evidence-based practices including mental health, school prevention programs, primary care interventions, substance use disorder treatments, and large state system initiatives; a “universal” tool has been developed for use of monitoring of general implementation strategies [50]. Results from studies using the SIC have repeatedly shown that when there is variability among the strategies sites use to implement a new practice, actions taken during the implementation process predict the achievement of program start-up and competency as measured with this tool [41, 51]. The SIC was tailored for this protocol using standardized SIC adaptation procedures [41] by the SIC developers, PrIMO model developers, and members of the research team. Consensus was reached regarding essential implementation activities for effective implementation fidelity for delivery of PrIMO, taking into consideration study start-up processes required by the Sponsor and activities that may occur through IF procedures. Study-specific milestones were mapped to the “universal” SIC to provide a low-burden observational tool to record progress of clinics over time as they implement PrIMO. Research Coordinators (RCs) at each of the sites will track and enter milestone completion dates into the web based SIC Tool.

#### Acceptability

Site staff and patient participants will complete longitudinal quantitative surveys and qualitative interviews (for a subset of participants) at various timepoints surrounding model launch (see Table 4) to assess acceptability. Site staff and patient participants will follow the same procedures for screening and enrollment: at the index (staff) or baseline (patients) visit, RCs will obtain verbal consent and conduct a brief screening to confirm study eligibility specific to the participant group (including an assessment of age, prisoner status, clinic role for site staff candidates, and length of exposure to the PrIMO model for patient candidates) before consenting potential participants. Site staff and patient participants will be compensated with $100 gift cards for each completed survey (up to six for site staff and three for patients), and $100 gift cards for the subset of participants that complete qualitative interviews (up to four for site staff and three for patients), though one site’s current union contract prohibits compensation for site staff. Staff and patient participants will self-complete quantitative survey assessments. After completing their first survey timepoint, participants will be invited to participate in qualitative interviews until participant maximums or saturation is reached. Participants will meet with research staff electronically (via Zoom) to review all significant elements of the qualitative interviews via an IRB-approved verbal consent. All study materials will be approved by the single IRB (sIRB, BRANY).

**Table 4:** Participant assessments

		3 months pre-PrIMO launch	Launch	3 months post-launch	6 months post-launch	9 months post-launch	12 months post-launch
Site staff participants							
The applied mental health research dissemination and implementation (AMHR) measure	Participant perceptions of model adoption, acceptability, appropriateness, feasibility, reach/access, organizational climate, leadership in implementing, and general leadership skills of the clinic	✓	✓	✓	✓	✓	✓
Modified clinical sustainability assessment tool (CSAT)	Participant perceptions of the model’s clinical sustainability		✓	✓	✓	✓	✓
The medical conditions regard scale (MCRS)	Biases, emotions, and expectations of chronic medical conditions, including Type2 diabetes, depressive disorder, HIV/AIDS, alcohol use disorder, stimulant use disorder, and opioid use disorder	✓	✓	✓	✓	✓	✓
Beliefs on MOUD	Prescriber perceptions of and barriers to utilizing MOUD (specifically buprenorphine, methadone, and naltrexone)	✓	✓	✓	✓	✓	✓
Implementation citizenship behavior scale (ICBS)	Identifies critical behaviors employees exhibit exceeding their expected role to support evidence-based practice implementation		✓^b^	✓^b^	✓^b^	✓^b^	✓^b^
Change rulers	Readiness and preparedness to implement PrIMO	✓	✓	✓	✓	✓	✓
Qualitative interviews^a^	Experiences providing care for patients with OUD, including use and knowledge (or lack) of the PrIMO model	✓	✓		✓		✓
Patient participants							
		Baseline (2 weeks post-exposure)		3 months post-baseline		6 months post-baseline	
Consumer AMHR	Participant perceived acceptability of model adoption, acceptability, appropriateness, feasibility, and reach/access	✓		✓		✓	
Substance use stigma mechanism scale (SU-SMS)	Self-reported feelings of internalized stigma	✓		✓		✓	
Qualitative interviews^a^	Experiences receiving care for OUD, including knowledge of the PrIMO model and experiences within the clinic	✓		✓		✓	

^a^For a subset of enrolled participants

^b^The champion provider and pharmacist will complete this measure rating other providers, team members, and pharmacy staff

As detailed in Table 4, site staff quantitative surveys will gather demographic characteristics and assess the acceptability of the PrIMO model implementation at the sites, including local partner perceptions of model implementation via the Applied Mental Health Dissemination and Implementation (AMHR) measure [52–55] and its clinical sustainability via a modified Clinical Sustainability Assessment Tool [56], biases of treating OUD via the Medical Conditions Regard Scale [57–59], perceived MOUD stigma over time via the Beliefs on MOUD assessment [60], and change rulers of readiness and preparedness to implement PrIMO over time. Site provider and pharmacist champions will also measure implementation citizenship, the behaviors that exceed expected tasks performed by employees at the site to support the implementation of the PrIMO model, via the Implementation Citizenship Behavior Scale (ICBS) [61–64].

Patient participant quantitative surveys will gather demographic characteristics and assess the perceived acceptability of the PrIMO model via the consumer-based AMHR, and feelings of internalized stigma from the Substance Use Stigma Mechanism Scale (SU-SMS) [65] over time.

Qualitative interviews with site staff will focus on exploring all five CFIR domains through key constructs related to MOUD barriers and facilitators, knowledge about PrIMO (self-evaluation), awareness of the needs of people with OUD, staff perspectives on the PrIMO model, staff perspectives on the process of implementing the PrIMO model, and staff perspectives on the PrIMO model in the context of the work environment. Qualitative interviews with patients will focus on exploring all CFIR domains (when possible) through key constructs related to knowledge about PrIMO, PrIMO experience/encounters with care team (Fidelity check), PrIMO experience/care team networks and communications (Fidelity check), care team attitudes/stigma, PrIMO experience/education (Fidelity check), patient needs and resources, and PrIMO model characteristics.

In addition to quantitative surveys and qualitative interviews, fidelity to the model and cost will also assist in determining the acceptability of the model’s implementation. Fidelity will be measured via a checklist developed by the study team that will systematically track how various team-based aspects of the PrIMO model are implemented, including those determined to be critical to the success of the model. Sites will be encouraged to complete the checklist during weekly clinical operations meetings (which is a core component of the PrIMO model). Checklist completion will help identify areas that may require targeted IF approaches to better support adherence to the PrIMO model as well as provide insight into how clinics tailor the model for location conditions (e.g., staffing structure, workflows, etc.). The Cost of Implementing New Strategies (COINS) is a method of mapping implementation resources that will collect information on the cost of implementing the PrIMO model, using the SIC. The COINS is not a measure of cost effectiveness but rather a way to map the cost of implementation resources using the SIC. The COINS tool has demonstrated success in identifying cost and resource differences between implementation strategies in studies using the SIC [66].

#### Impact

Treatment outcomes, including a site’s capacity for MOUD, will be assessed via EHR data extracted from the clinic systems. EHR records will be extracted in coordination with a Data Dictionary developed specifically for the use of this study, which detail the fields each site will be asked to extract. At a minimum, the study will extract EHR data to explore treatment outcomes for patients on MOUD at the clinic (i.e., average retention, MOUD adherence, and toxicology including opioid abstinence), and capacity for treating patients with MOUD (i.e., the number of patients on MOUD, providers prescribing MOUD, and MOUD patients per prescribing provider) at all sites. EHR data will anchor on the launch of the model, allowing comparison of the model’s impact on these fields from 1-year pre-PrIMO launch to 1-year post-PrIMO launch at each site.

#### Process measures

Process measures will identify areas of attention for IF procedures and implementation support. These include qualitative interview matrices, log of IF activities at each site (e.g., meetings and learning collaborative attendance, rollout of promotional material, dates of academic detailing, etc.), change rulers to assess readiness and preparedness to provide PrIMO (delivered alongside all site staff participant assessments), and focus groups as part of the formative evaluation. Approximately two focus groups will be conducted (one with supervisors/leadership and one with supervisees/staff) at each site by the external facilitators. Focus groups will be audio recorded to ensure the accuracy of information conveyed to the investigators and clinic teams implementing the model. Demographic information will be collected to characterize participating staff. Focus group findings are not considered outcome measures, and therefore all learning they generate will inform the process for implementation at each site.

## Analysis

The primary outcome measure will result in a binary variable that represents whether the site completes Stage 6 (model launch) of the SIC. The summary statistics for this outcome will include the number and percentage (%) of sites reaching Stage 6. SIC variables of duration (length of time in days) and proportion scores will be treated as continuous variables and summarized using descriptive statistics [median, mean, standard deviation (SD), range and quartiles] and comparisons will be made between study sites to understand the most optimal patterns of implementing PrIMO. Although the sample of four feasibility sites is insufficient to draw statistical conclusions, activity completion and site implementation behavior will be observed across the pre-implementation and implementation phases.

Combining quantitative site staff and patient perspectives of model acceptability with qualitative interview, model fidelity, and cost data will serve to contextualize and better understand the acceptability of the PrIMO model at the primary care clinics engaged in the study. The secondary outcomes (implementing PrIMO model acceptability and impact) will result in quantitative and qualitative endpoints. Quantitative analyses at the site level will be purely descriptive in nature and precision estimates for key secondary outcomes at the staff, provider and patient level will be conducted. Categorical outcomes collected as Likert scale items will be presented as ordinal variables (as appropriate). Responses with two non-ordinal levels such as Yes/No or Complete/Incomplete, will be analyzed as binary variables using summary statistics such as frequency/counts and proportions. Analyses for quantitative secondary outcomes will use the same descriptive statistics described in the primary outcome analysis and will be presented by timepoint. Corresponding 95% confidence intervals will be provided for key secondary outcomes. This is an exploratory study and will not require multiplicity adjustments for multiple testing. Any exploratory analyses that use hypothesis testing will use an alpha level of 0.05 for evaluating statistical significance.

Qualitative interviews will undergo direct content analysis [67] to identify themes and subthemes found across participant subgroups. Analysis of these outcomes will be conducted at individual data collection time points (site summary matrices populated synchronously by time point) and at study completion (time-ordered, sequential site summary matrices populated diachronously).

Interviewers will use individual interview forms (i.e., ‘templated summaries’ [68]) as the analytic tools for the cross-sectional analyses, which will be populated as soon as possible following each interview. Interviewers will embed relevant text segments from interview transcripts generated by Zoom into interview forms that have been pre-coded by CFIR constructs. A secondary analyst (i.e., member of the team who did not conduct the interview) will review completed interview forms for thoroughness and accuracy. Once all interviews have been conducted at a site for a given data collection timepoint, a rapid cross-sectional analysis will be conducted for each site (site staff and patients), generating site summary matrices by timepoint (a random subset will be reviewed by secondary analysts to ensure trustworthiness)**.** Mixed method analysis, or the systematic integration of qualitative and quantitative measures of acceptability for the purposes of triangulation (seeking convergence/divergence of both types of outcome data) and expansion (seeking a more comprehensive understanding of acceptability of PrIMO and its implementation) [69, 70] will occur at study completion once all data have been collected.

The study hypothesizes that it will be feasible to implement the PrIMO model (complete SIC Stage 6) using the IF implementation approach, and that it will be acceptable, will positively impact patient outcomes one year post-model launch (e.g., increased MOUD treatment retention, medication regimen adherence, service utilization for co-morbid conditions, and decreased substance use including opioid abstinence), and will increase each clinic’s capacity for caring for patients with MOUD (e.g., increased number of patients, number of prescribers, and rate of patients per prescriber).

## Discussion and next steps

Through a longitudinal, mixed-methods approach, we will assess the feasibility, acceptability, and impact of implementing this model of care for OUD in four diverse primary care settings. As this study will examine implementation in situ, real-world problems faced by each clinic (e.g., a healthcare staffing shortage) will directly impact results and be translatable to other clinics that may want to implement PrIMO. These data may be used to inform how PrIMO may be implemented in primary care settings to help providers treat OUD, increase access to and retention in MOUD, enhance the role of pharmacists, and reduce stigmatization of MOUD (for providers and patients) with the goal of decreasing the morbidity and mortality of OUD in the US.

As the opioid overdose epidemic continues to claim lives at an alarming rate, multidisciplinary approaches to support both patients receiving MOUD and prescribers are needed. Leveraging relationships between primary care clinics, pharmacists and pharmacies might improve access and outcomes. As the role of pharmacists continues to expand to support chronic disease management for many conditions, there is tremendous opportunity to engage pharmacists in integrated care teams to provide MOUD. The PrIMO model of care offers a patient-centered approach to MOUD that integrates the pharmacist as a key member of the care team. Stigma related to OUD is a pervasive barrier in health systems that inhibits wider implementation of MOUD treatment and scalability [71]. Lessons learned in this implementation feasibility study may inform recommendations to increase knowledge of OUD as a chronic disease that includes safe and effective medication treatment, enhances trust and rapport among care team members to increase confidence and mitigate burnout, and bolsters the relationship between providers and patients to help promote desired treatment outcomes.

One of the novel aspects of this study is that its design will allow us to share practical learnings as they emerge. Presentations to clinic systems, pharmacy groups, substance use treatment authorities, training programs, and other venues can widely disseminate lessons learned from this study in nontraditional ways. These dissemination efforts could have a novel impact on systems throughout the US that could help to rapidly scale this model. These efforts could also inform policies related to access to care, including those related to reimbursement strategies to make models like PrIMO easier for clinics to implement and sustain.

## Data Availability

Once complete, data from this study protocol will be available on the NIDA CTN Data Share website.
